# Identification of key signaling pathways and hub genes related to immune infiltration in Kawasaki disease with resistance to intravenous immunoglobulin based on weighted gene co-expression network analysis

**DOI:** 10.3389/fmolb.2023.1182512

**Published:** 2023-05-30

**Authors:** Yue Wang, Yinyin Cao, Yang Li, Meifen Yuan, Jin Xu, Jian Li

**Affiliations:** ^1^ Clinical Laboratory Center, Children’s Hospital of Fudan University, National Children’s Medical Center, Shanghai, China; ^2^ Cardiovascular Center, Children’s Hospital of Fudan University, National Children’s Medical Center, Shanghai, China

**Keywords:** IVIG-resistance, intravenous immunoglobulin, Kawasaki disease, weighted gene coexpression network analysis, immune infiltration, neutrophil

## Abstract

**Background:** Kawasaki disease (KD) is an acute vasculitis, that is, the leading cause of acquired heart disease in children, with approximately 10%–20% of patients with KD suffering intravenous immunoglobulin (IVIG) resistance. Although the underlying mechanism of this phenomenon remains unclear, recent studies have revealed that immune cell infiltration may associate with its occurrence.

**Methods:** In this study, we downloaded the expression profiles from the GSE48498 and GSE16797 datasets in the Gene Expression Omnibus database, analyzed differentially expressed genes (DEGs), and intersected the DEGs with the immune-related genes downloaded from the ImmPort database to obtain differentially expressed immune-related genes (DEIGs). Then CIBERSORT algorithm was used to calculate the immune cell compositions, followed by the WGCNA analysis to identify the module genes associated with immune cell infiltration. Next, we took the intersection of the selected module genes and DEIGs, then performed GO and KEGG enrichment analysis. Moreover, ROC curve validation, Spearman analysis with immune cells, TF, and miRNA regulation network, and potential drug prediction were implemented for the finally obtained hub genes.

**Results:** The CIBERSORT algorithm showed that neutrophil expression was significantly higher in IVIG-resistant patients compared to IVIG-responsive patients. Next, we got differentially expressed neutrophil-related genes by intersecting DEIGs with neutrophil-related module genes obtained by WGCNA, for further analysis. Enrichment analysis revealed that these genes were associated with immune pathways, such as cytokine-cytokine receptor interaction and neutrophil extracellular trap formation. Then we combined the PPI network in the STRING database with the MCODE plugin in Cytoscape and identified 6 hub genes (TLR8, AQP9, CXCR1, FPR2, HCK, and IL1R2), which had good diagnostic performance in IVIG resistance according to ROC analysis. Furthermore, Spearman’s correlation analysis confirmed that these genes were closely related to neutrophils. Finally, TFs, miRNAs, and potential drugs targeting the hub genes were predicted, and TF-, miRNA-, and drug-gene networks were constructed.

**Conclusion:** This study found that the 6 hub genes (TLR8, AQP9, CXCR1, FPR2, HCK, and IL1R2) were significantly associated with neutrophil cell infiltration, which played an important role in IVIG resistance. In a word, this work rendered potential diagnostic biomarkers and prospective therapeutic targets for IVIG-resistant patients.

## 1 Introduction

Kawasaki disease (KD), also known as mucocutaneous lymph node syndrome, is an acute febrile systemic vasculitis that mainly occurs in children under five (Agarwal and Agrawal, 2017). The pathogenesis of KD remains unknown, but an immune-mediated inflammatory cascade triggered by an unknown stimulus in genetically susceptible children is believed to be one of the major mechanisms (Rife and Gedalia, 2020). The most severe complication of KD is the occurrence of coronary artery lesions, such as coronary artery aneurysms, which is the most common cause of acquired heart disease among children in developed countries (Hedrich et al., 2018). Early use of high-dose intravenous immunoglobulin (IVIG) is well-accepted as the standard treatment for KD (Fukui et al., 2021). The risk of coronary artery aneurysms will be reduced five-fold if IVIG is used within 10 days of fever onset (Newburger et al., 2016). However, up to 20% of KD patients are IVIG-resistant and have a persistent or recrudescent fever at least 36 h after the end of the initial IVIG infusion (Kaya Akca et al., 2022). These patients have a higher risk of developing coronary artery lesions, compared to IVIG responders (Song, 2019). Therefore, it is crucial to explore new biomarkers to predict KD patients’ treatment response, which can improve the prediction of prognosis and guide clinical decision-making.

In recent years, to better understand the mechanisms underlying IVIG resistance in KD and identify new treatment options, numerous studies have focused on the relationship between immunity and IVIG resistance. For instance, one study involving T cells has suggested that excessive CD8^+^ T cell activation and the imbalance between CD8^+^ T cell activation and inhibition contribute to the pathogenesis of KD. While IVIG can inhibit CD8^+^ T cell activation, excessive activation of these cells may lead to IVIG resistance (Ye et al., 2016). Additionally, research involving neutrophils has found that IVIG-resistant patients have a higher percentage of neutrophils and higher neutrophil-to-lymphocyte ratios than IVIG-responsive patients at IVIG administration (Sato et al., 2013; Lee and Song, 2016; Han et al., 2022). These studies provide new insights into the importance of immune regulation in IVIG resistance.

**FIGURE 1:**
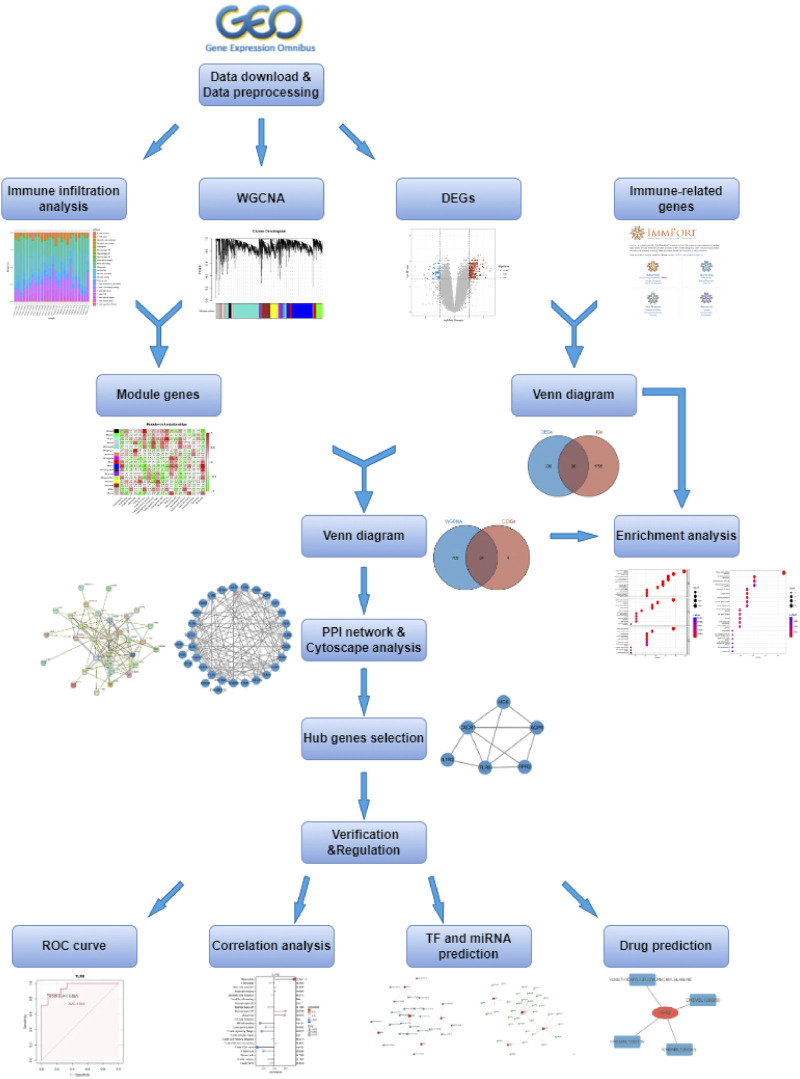
The flowchart of this study.

Weighted gene co-expression network analysis (WGCNA) is a systems biology approach that studies patterns of gene co-expression and constructs co-expression network models based on gene expression profiles. This method has been widely used to investigate the pathogenesis of many diseases, including KD (Wang T et al., 2022). In this study, we used WGCNA to identify module genes highly associated with immune cell infiltration, to better understand the development of IVIG resistance, and make it possible to design early diagnosis and therapeutic procedures for IVIG resistance. The workflow diagram of this study was displayed in Figure 1.

## 2 Materials and methods

### 2.1 Data acquisition

This study utilized two datasets, namely GSE48498 and GSE16797, obtained from the Gene Expression Omnibus (GEO) database (http://www.ncbi.nlm.nih.gov/geo) (Ogihara et al., 2009; Ogihara et al., 2014). Both datasets were derived from the same platform GPL570, which was important for merging the data and performing subsequent integrated analysis. Then a total of 26 KD patients were included in this study, of which 14 patients were resistant to IVIG treatment and 12 patients were responsive to IVIG treatment (Table 1). Additionally, 2483 immune-related genes (IGs) data were retrieved from the ImmPort database (https://immport.niaid.nih.gov), which would be used in our study (Bhattacharya et al., 2018).

**TABLE 1:** IVIG-resistant and IVIG-responsive KD patients.

Platform	Datasets	Total samples	Intravenous immunoglobulin G (IVIG)	
			Groups	Samples
GPL570	GSE48498	28	IVIG-resistant	GSM1179961
				GSM1179962
				GSM1179963
				GSM1179964
				GSM1179965
				GSM1179966
				GSM1179967
				GSM1179968
			IVIG-responsive	GSM1179983
				GSM1179984
				GSM1179985
				GSM1179986
				GSM1179987
				GSM1179988
	GSE16797	34	IVIG-responsive	GSM421900
				GSM421902
				GSM421904
				GSM421906
				GSM421908
				GSM421910
			IVIG-resistant	GSM421912
				GSM421914
				GSM421916
				GSM421918
				GSM421920
				GSM421922

### 2.2 Data preprocessing and screening of differentially expressed genes

After the raw data of the GSE48498 and GSE16797 were read by the “Affy” package of Bioconductor (http://www.bioconductor.org/packages/release/bioc/html/affy.html) in R software, they were performed with background correction, normalization, and probe summarization using robust multi-array average (RMA) algorithm (Gautier et al., 2004; Harbron et al., 2007). In addition, the batch effect was eliminated by utilizing the combat function of the R software package “SVA” (Leek et al., 2012). The effect of data correction was demonstrated using two-dimensional PCA cluster plots before and after data correction respectively (Metsalu and Vilo, 2015).

Next, the differentially expressed genes (DEGs) between the IVIG-resistant group and the IVIG-responsive group were identified using the “Limma” package in R software (Diboun et al., 2006). The filter criteria for DEGs were set as |log2 fold change (FC)| > 1 and adjusted *p*-value < 0.05. The volcano plot and heatmap were drawn using the “ggplot2” package in R software to visualize the DEGs. Differentially expressed immune-related genes (DEIGs) were obtained by taking the intersection of DEGs and IGs using the “Venndiagram” R package (Chen and Boutros, 2011).

### 2.3 GO and KEGG enrichment analysis

Gene Ontology (GO) annotation and Kyoto Encyclopedia of Genes and Genomes (KEGG) enrichment analysis were performed using the “clusterProfiler” R package to reveal the potential biological functions and pathway mechanisms of genes (Yu et al., 2012). Adjusted *p* < 0.05 was considered statistically significant in this study.

### 2.4 Immune infiltration analysis

A bioinformatics algorithm called CIBERSORT (https://cibersortx.stanford.edu/) was used to measure the infiltration status of immune cells to quantify the relative proportions of infiltrating immune cells from the gene expression profiles of our samples (Newman et al., 2015). A standard set of 22 kinds of immune cell subsets (IM22) having 1,000 permutations were utilized to assess the predicted abundance level of immune cells. To visualize the results of the CIBERSORT algorithm, we used the “pheatmap” package to generate a heatmap about the proportion of 22 infiltrating immune cells in each sample, and the “ggboxplot” package to obtain a boxplot to show the differences in each infiltrating immune cell between the IVIG-resistant group and IVIG-responsive group, respectively.

### 2.5 Construction of weighted gene Co-expression network

A gene co-expression network was constructed using the WGCNA method, which was implemented with the R-based package “WGCNA” (Langfelder and Horvath, 2008). First, we selected the top 5000 genes based on variance from the gene expression data for analysis, then performed hierarchical clustering analysis to detect and remove outlier samples. In order to construct a scale-free network, the optimal soft-thresholding power was identified and the adjacency matrix was transformed into a topological overlap matrix (TOM) (Shuai, et al., 2021). Subsequently, the hierarchical cluster tree was cut into gene modules using the dynamic tree cut algorithm, with a minimum module size of 30 genes. Then, the heatmap was plotted to reflect the relationships between each module and 18 subtypes of immune infiltrating cells, and the module with a high correlation coefficient was selected. Finally, the R package “Venndiagram” was used to take the intersection of these genes in the selected module and DEIGs, and the obtained genes were used for subsequent analysis.

### 2.6 Construction of protein-protein interaction network and identification of hub genes

The above-obtained genes were subjected to protein-protein interaction (PPI) network analysis using the Search Tool for the Retrieval of Interacting Genes/Proteins (STRING, https://string-db.org/) database (Szklarczyk et al., 2017). Moreover, Cytoscape is an open-source software project for integrating biomolecular interaction networks, which is one of the most powerful network biology analyses and visualization tools when applied in conjunction with large databases of protein-protein, protein-DNA, and genetic interactions (Shannon et al., 2003). Thus, we visualized the PPI network through Cytoscape software. Additionally, the Cytoscape plugin Molecular Complex Detection (MCODE), an app for clustering a given network based on its topology to find densely connected regions (Bandettini et al., 2012), was utilized to identify clusters of genes that were highly connected across the entire PPI network, which ultimately led to the determination of hub genes.

### 2.7 ROC curve analysis

Receiver operating characteristic (ROC) curves are standard statistical tools for the analysis of disease markers and the area under a ROC curve (AUC) is a popular measure of diagnostic accuracy that is independent of the proportion of diseased subjects in the analyzed sample (Parodi et al., 2022). We separately performed ROC curve analysis on each screened hub gene to verify its accuracy in GSE48498 and GSE16797 two datasets. ROC curve analysis was executed with the R package “pROC” (Robin et al., 2011). The AUC value was calculated to evaluate the predictive utility of these hub genes. When the AUC>0.7, the hub gene was considered to have good sensitivity for IVIG resistance diagnosis.

### 2.8 Correlation analysis between diagnostic genes and infiltrating immune cells

Immune infiltration analysis was performed using the CIBERSORT algorithm. Subsequently, Spearman correlation analysis was employed to examine the relationship between diagnostic gene expression and immune cell infiltration (Pripp, 2018). To illustrate these relationships more intuitively, lollipop plots were created using the “ggplot2” package for visualization.

### 2.9 Construction of potential TF-, MiRNA-, and drug-diagnostic gene regulatory network

NetworkAnalyst (http://www.networkanalyst.ca/) is a comprehensive web-based tool that integrates all three steps of biological network analysis - identification of genes or proteins of interest, network construction, and network analysis and visualization - and is designed to allow researchers to perform a variety of common and complex meta-analyses of gene expression data through an intuitive web interface (Zhou et al., 2019). To identify possible transcription factors (TFs) and miRNA regulating diagnostic genes, we used NetworkAnalyst online database integrating TF database ENCODE (https://www.encodeproject.org/) and miRNA database miRTarBase (https://mirtarbase.cuhk.edu.cn/) (ENCODE Project Consortium, 2011; Huang et al., 2022). What’s more, the Drug-Gene Interaction Database (DGIdb; https://www.dgidb.org/) integrates, organizes, and presents information on drug-gene interactions and genetic drug-forming properties from papers, databases, and web resources (Cotto et al., 2018). And the DGIdb online database was used to predict the potential targeted drugs that interacted with the diagnostic genes, then the drug-gene interaction network was visualized in the Cytoscape software.

### 2.10 Statistical analysis

All statistical analyses in our study were performed with the R software (version 4.2.2, https://www.r-project.org/). Unless otherwise specified, the *p*-value for statistical significance was set at 0.05.

## 3 Results

### 3.1 Identification of DEGs

**FIGURE 2:**
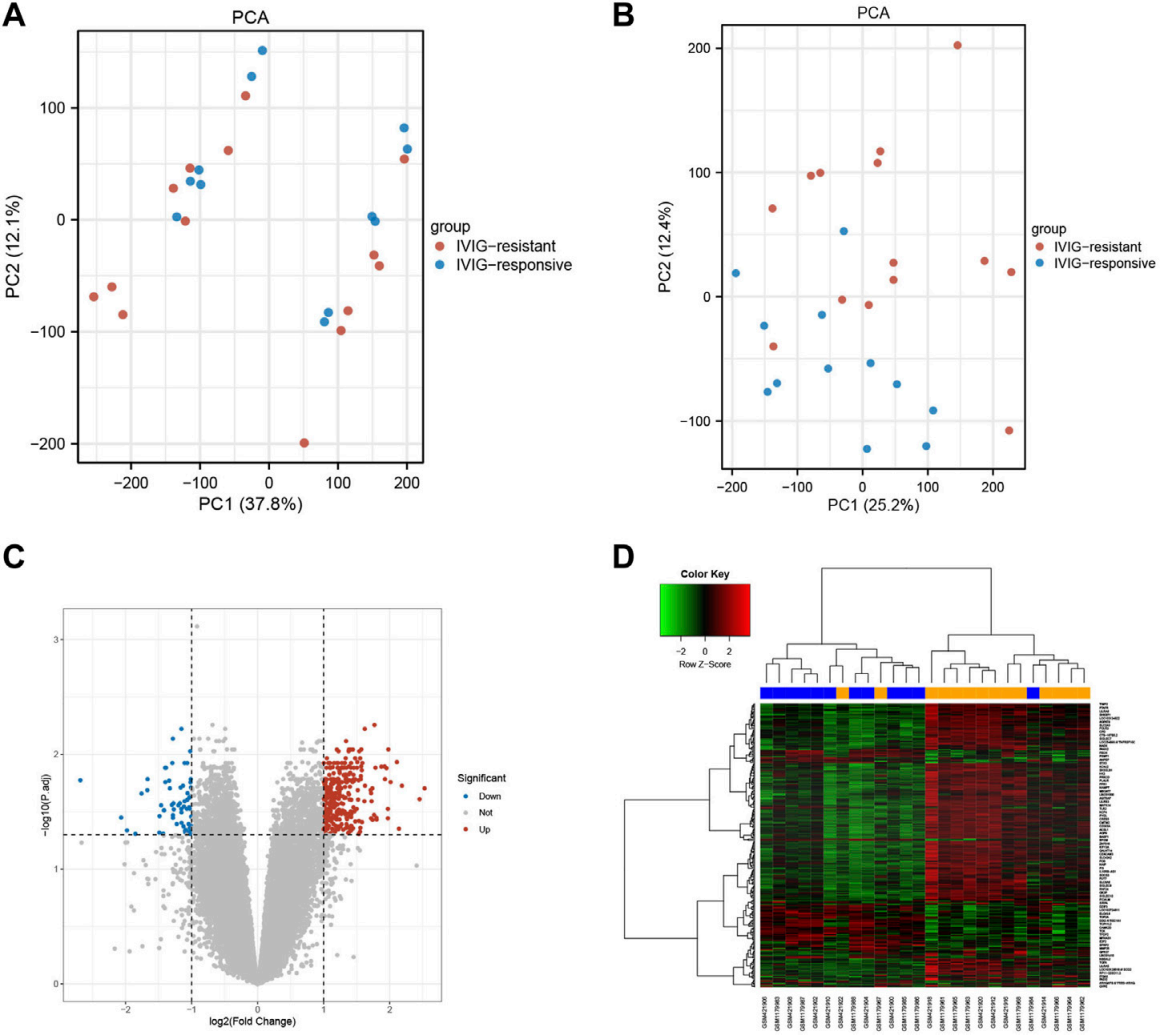
The effects of data processing and the recognition of differences express genes. **(A)** PCA diagram before data processing. **(B)** PCA diagram after data processing. **(C)** The volcano plot of DEGs. The upregulated genes were marked in red dots, while the downregulated genes were marked in blue dots. **(D)** The heatmap of DEGs. The upregulated genes were represented by red, and the downregulated genes were represented by green.

The selected samples from the GSE48498 and GSE16797 datasets were subjected to background correction, normalization, and batch removal. The sample distributions before and after the removal of batch effects were visualized in two-dimensional PCA cluster diagrams (Figures 2A, B). All samples in Figure 2A were divided into two distinct clusters, however, Figure 2B showed that after processing, the samples in the two datasets were more evenly mixed, indicating a significant effect of batch removal and more reliable integrated data. Differential gene expression analysis was performed using the processed data, and a total of 246 DEGs were obtained (| log2 FC |> 1 and adjusted *p* < 0.05), of which 189 DEGs were upregulated and 57 were downregulated (Supplementary Table S1). The volcano plot and heatmap of DEGs were shown respectively in Figures 2C, D.

### 3.2 Functional enrichment analysis of DEIGs

**FIGURE 3:**
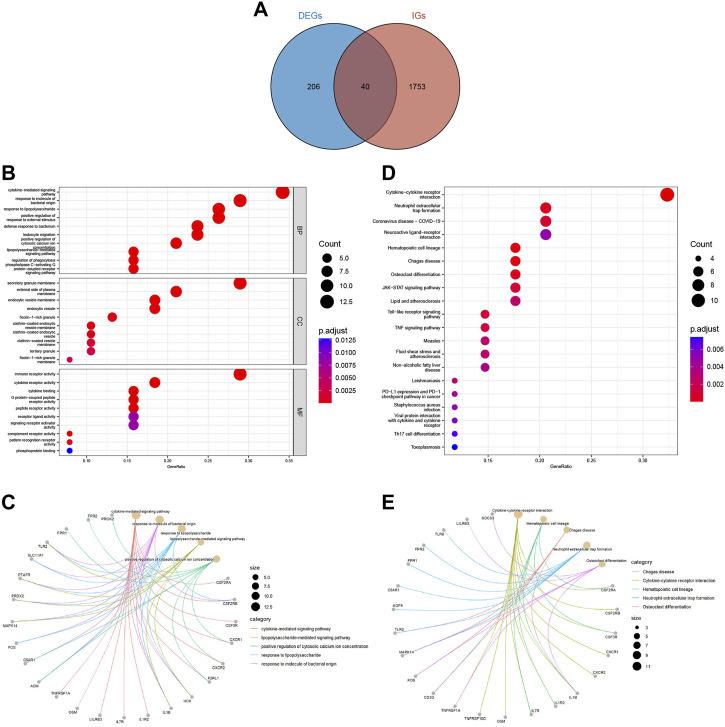
Acquisition and functional enrichment analysis of DEIGs. **(A)** Venn diagram of DEGs and IGs. **(B)** GO enrichment bubble chart of DEIGs. **(C)** GO pathway-gene network diagram of DEIGs. **(D)** KEGG enrichment bubble chart of DEIGs. **(E)** KEGG pathway-gene network diagram of DEIGs.

We downloaded 2483 IGs from the ImmPort database and obtained 40 differentially expressed immune-related genes (DEIGs) through an intersection with 246 DEGs (Supplementary Table S2). A Venn diagram was used to show the intersection between the IGs and DEGs (Figure 3A). GO analysis of DEIGs indicated that, for the biological process (BP), these genes were mainly involved in cytokine-mediated signaling pathways, response to bacterial-origin molecules, and response to lipopolysaccharides. In terms of cellular component (CC), DEIGs may play critical roles in secretory granule membranes, plasma membrane outer and inner vesicle membranes. Regarding molecular function (MF), DEIGs were found to have crucial roles in immune receptor activity, cytokine receptor activity, and cytokine binding (Figures 3B, C). In addition, KEGG pathway enrichment analysis revealed that DEIGs were mainly enriched in cytokine-cytokine receptor interactions, neutrophil extracellular trap (NET) formation, and coronavirus disease - COVID-19 (Figures 3D, E).

### 3.3 Immune infiltration analysis

**FIGURE 4:**
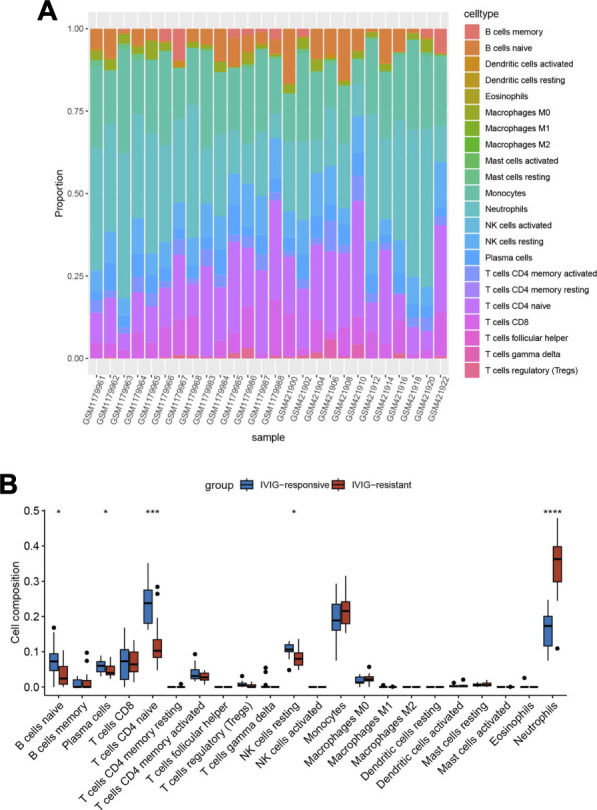
Immune infiltration analysis based on the CIBERSORT algorithm. **(A)** The heatmap of the enrichment fraction of 22 types of infiltrating immune cells in each sample. **(B)** The box plot of 22 types of immune infiltrating cells in the IVIG-resistant group and the IVIG-responsive group.

To explore the immune microenvironment of IVIG-resistant patients, we estimated the relative abundance of 22 types of immune cells using the CIBERSORT algorithm. Figure 4A showed the composition of the 22 types of immune infiltration cells in each sample. Figure 4B compared the infiltration differences of 22 types of immune cells between the IVIG-resistant group and the IVIG-responsive group. The results indicated that naïve CD4 T cells and neutrophils were significantly different between the two groups, with the most significant difference observed in neutrophil infiltration.

### 3.4 Construction of weighted gene Co-expression network

**FIGURE 5:**
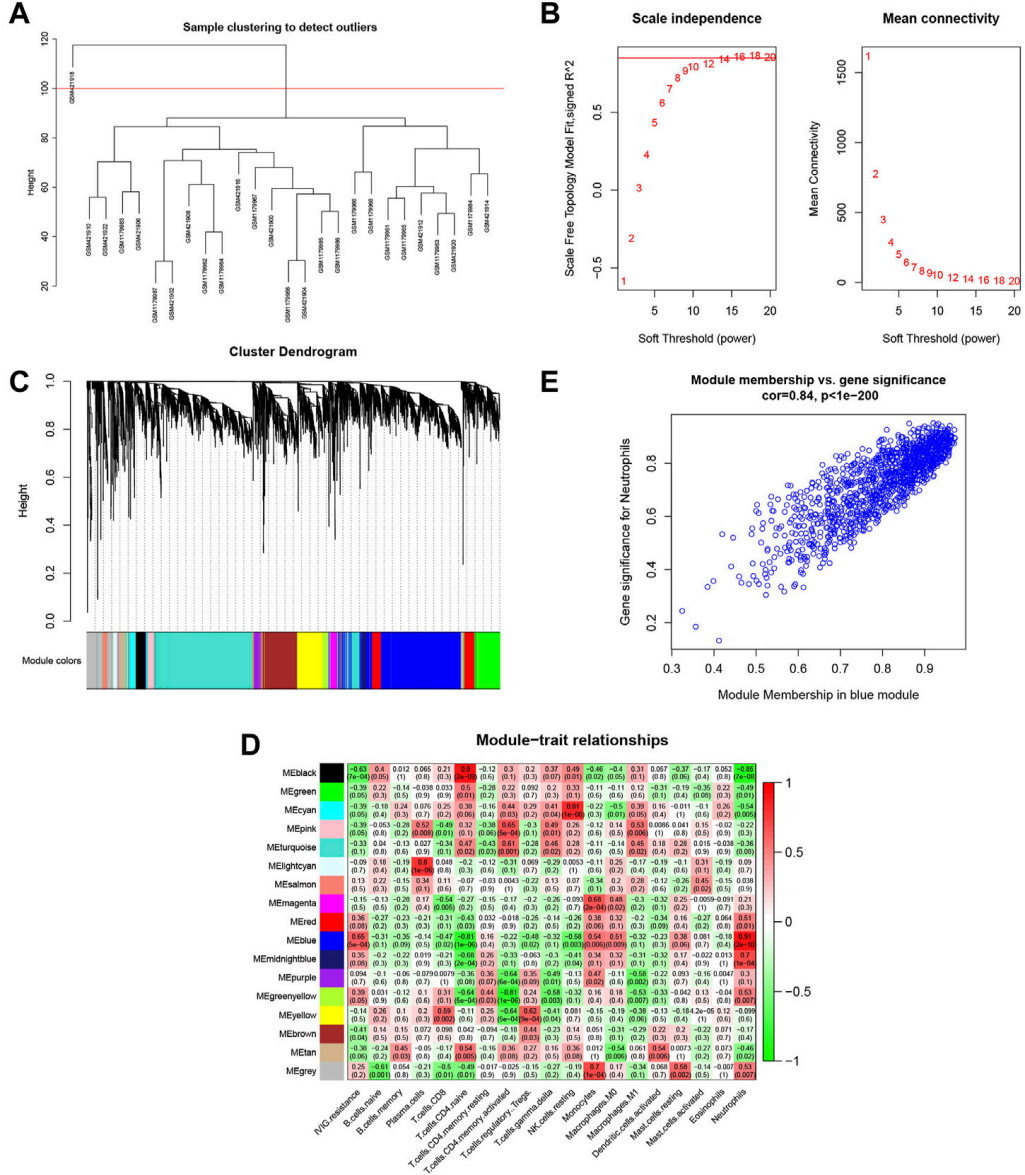
Identification of the gene modules related to infiltrating immune cells by WGCNA. **(A)** Sample clustering of WGCNA to detect abnormal samples. **(B)** Selection of soft-thresholding power. The left panel displayed the change of the fitting index of different soft-thresholding powers; the right panel displayed the mean connectivity corresponding to different soft-thresholding powers. **(C)** Hierarchical clustering dendrogram of identified co-expressed genes. Different colors reflected the corresponding modules, and the gray module indicated that the genes were not assigned to any module. **(D)** The heatmap of the relationship between each gene module and each immune cell. The red represented a positive correlation, while the green represented a negative correlation. **(E)** Scatterplot of gene significance (GS) for neutrophil *vs*. module membership (MM) in the blue modules.

In Figure 5A, the sample clustering tree showed that one outlier was removed, and 25 samples were retained, of which 13 belonged to the IVIG-resistant group and 12 belonged to the IVIG-responsive group. We then selected the optimal soft-thresholding power of 16 to ensure that our gene distribution conformed to a scale-free network (Figure 5B). Next, WGCNA analysis yielded the gene dendrogram and module colors and identified 17 modules in this study (Figure 5C). After analyzing the correlation of each module with the 22 infiltrating immune cells, the blue module was found to possess the highest positive correlation with neutrophils and presented the highest score of total scores in the plot of the module-trait relationship (Figure 5D). Figure 5E illustrated the significant correlation (cor = 0.84, P < 1e-200) between gene significance (GS) and module membership (MM) in the blue module, indicating that the genes highly associated with neutrophils were also important elements in this blue module, thus they were suitable for further analysis.

### 3.5 Functional enrichment analysis of DENGs

**FIGURE 6:**
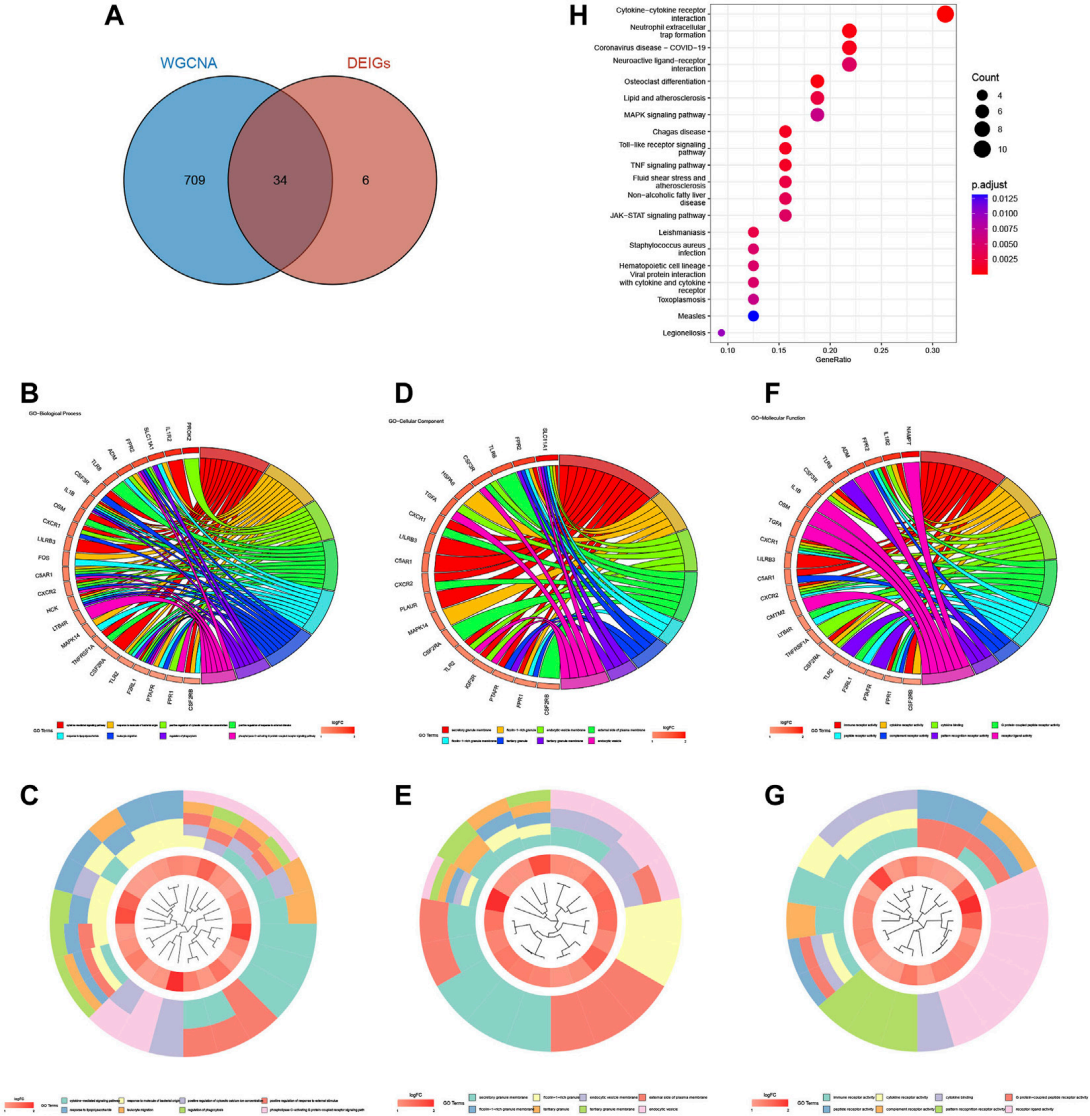
Acquisition and enrichment analysis of DENGs. **(A)** Venn diagram of the blue module genes and DEIGs. **(B, C)** GO chord diagram and GO cluster diagram of BP of DENGs. **(D, E)** GO chord diagram and GO cluster diagram of CC of DENGs. **(F, G)** GO chord diagram and GO cluster diagram of MF of DENGs. **(H)** The KEGG enrichment bubble chart of DENGs.

A total of 743 genes were contained in the blue module (Supplementary Table S3). Through the intersection between genes in the blue module and DEIGs, we identified 34 differentially expressed neutrophil-related genes (DENGs) (Figure 6A). GO and KEGG analyses were performed to explore relevant molecular biological functions and pathways of these 34 DENGs. GO analysis showed that the BPs of these genes were mainly involved in cytokine-mediated signaling pathways, response to bacterial-origin molecules, and positive regulation of cytosolic calcium ion concentration (Figures 6B, C). The CCs of these genes were mostly in the area of secretory granule membrane, lactotransferrin-1-containing granule, and endocytic vesicle membrane (Figures 6D, E). The MFs of these genes were significantly enriched in immune receptor activity, cytokine receptor activity, and cytokine binding (Figures 6F, G). KEGG analysis indicated that the 34 DENGs were significantly related to immune pathways, such as cytokine-cytokine receptor interaction, neutrophil extracellular trap formation, and coronavirus disease - COVID-19 (Figure 6H).

### 3.6 Construction of protein-protein interaction network and identification of hub genes

**FIGURE 7:**
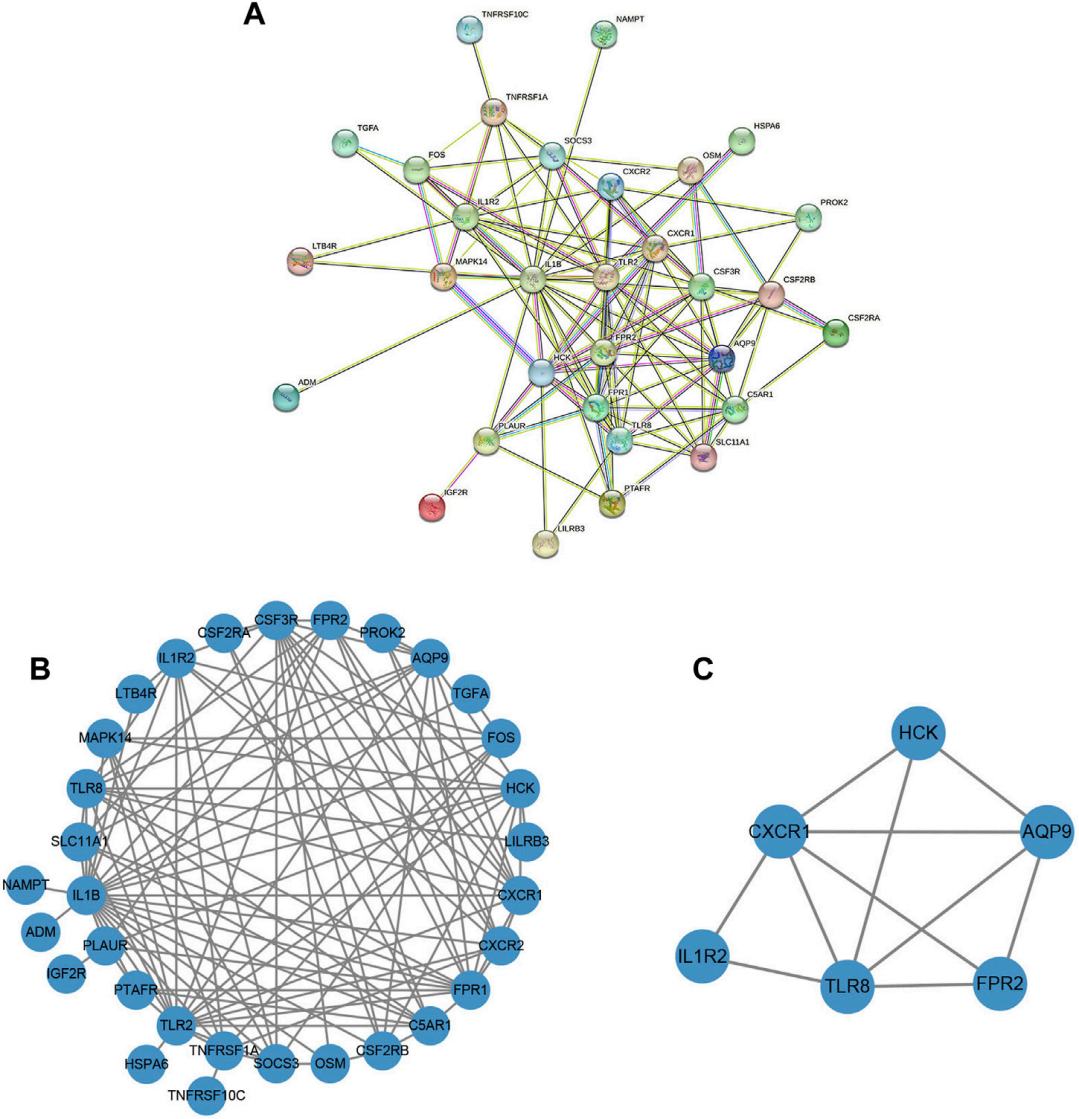
The construction of the PPI network and the identification of the hub genes. **(A)** PPI network based on the STRING database. **(B)** The PPI network was visualized by Cytoscape software. **(C)** Screening of clusters of genes that were highly connected using the MCODE algorithm.

A protein-protein interaction (PPI) network of the obtained DENGs was constructed by the STRING database (Figure 7A). We visualized the PPI network through Cytoscape software and identified hub genes in the PPI network using the MCODE plugin (Figures 7B, C). Finally, six hub genes were identified, namely TLR8, CXCR1, HCK, AQP9, FPR2, and IL1R2.

### 3.7 ROC curve analysis

**FIGURE 8:**
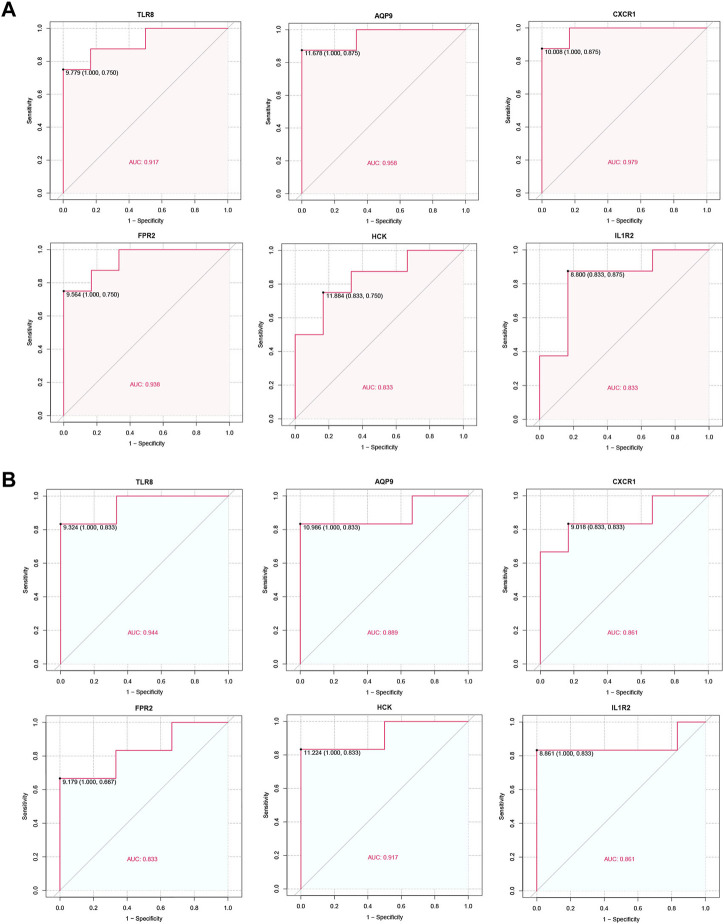
ROC curve of the hub genes. **(A)** ROC curve of hub genes in the GSE48498. **(B)** ROC curve of hub genes in the GSE16797.

The diagnostic performance of the identified hub genes for IVIG resistance was evaluated by ROC curve analysis in GSE48498 and GSE16797 two datasets separately (Figures 8A, B). We considered an AUC > 0.7 as the screening criterion for hub genes. As a result, in the GSE48498 dataset, six hub genes had AUC > 0.7, with TLR8, AQP9, CXCR1, and FPR2 having AUC > 0.9. In the GSE16797 dataset, six hub genes also had AUC > 0.7, with only TLR8 and HCK having AUC > 0.9. Altogether these results suggested that six hub genes were able to distinguish IVIG-resistant from IVIG-responsive patients after KD patients were treated with IVIG in both datasets, so we believed that they could have good diagnostic value and were considered as potential biomarkers for the diagnosis of IVIG resistance.

### 3.8 Correlation analysis between diagnostic genes and immune cells

**FIGURE 9:**
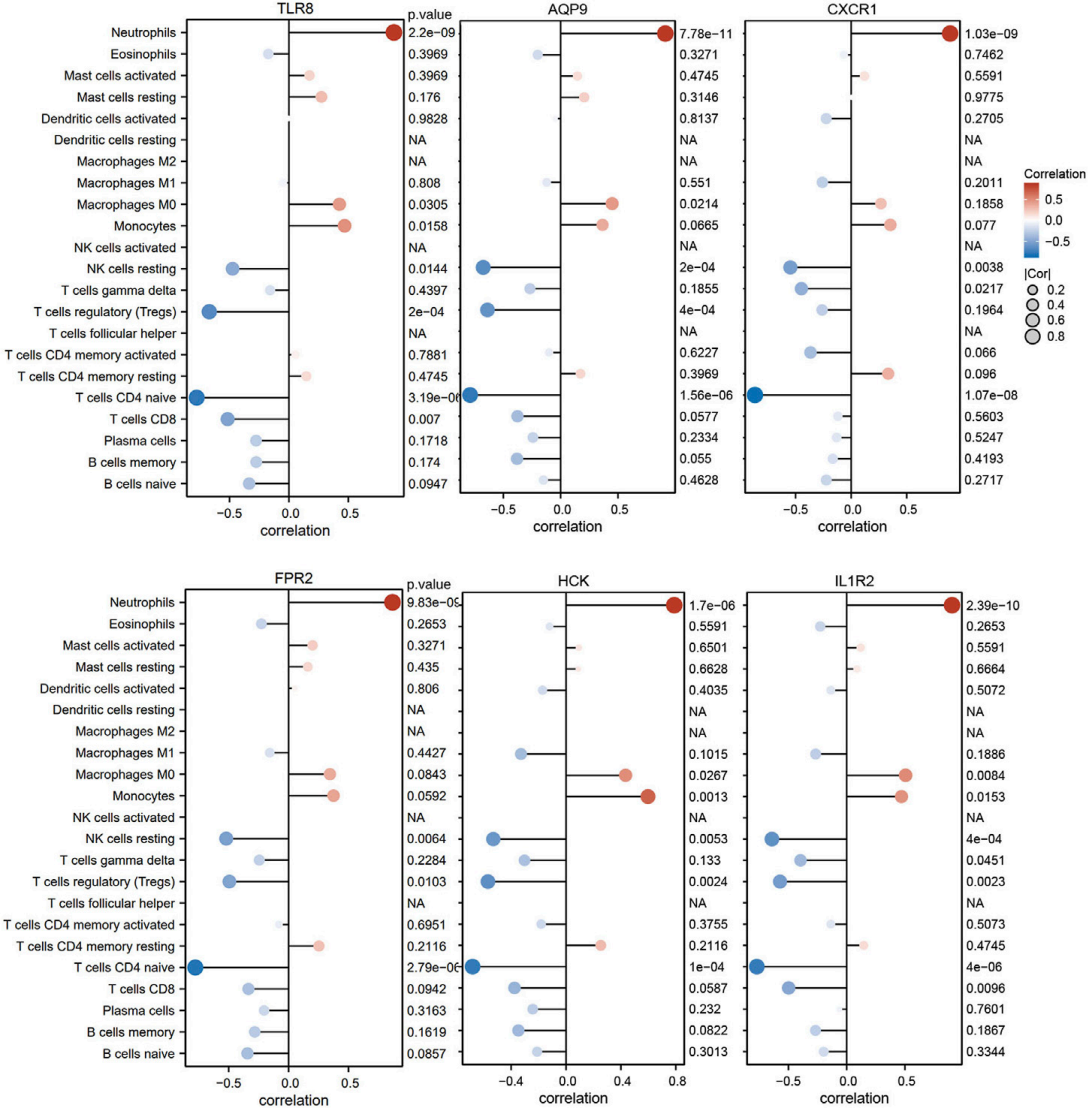
The correlation between diagnostic genes and infiltrating immune cells.

For the purpose of better understanding the role of six diagnostic genes in immune infiltration, we performed Spearman’s correlation analysis to determine the correlation of hub genes with infiltrating immune cells. The results showed that six diagnostic genes, including TLR8, AQP9, CXCR1, FPR2, HCK, and IL1R2, were significantly positively correlated with neutrophil infiltration, which could confirm that they were screened from the blue module related to neutrophils. In addition, these diagnostic genes were negatively correlated with the immune infiltration of naïve CD4 T cells, resting NK cells, and regulatory T cell (Figure 9).

### 3.9 Prediction of key TF-, MiRNA-, and drug-diagnostic gene regulatory network

**FIGURE 10:**
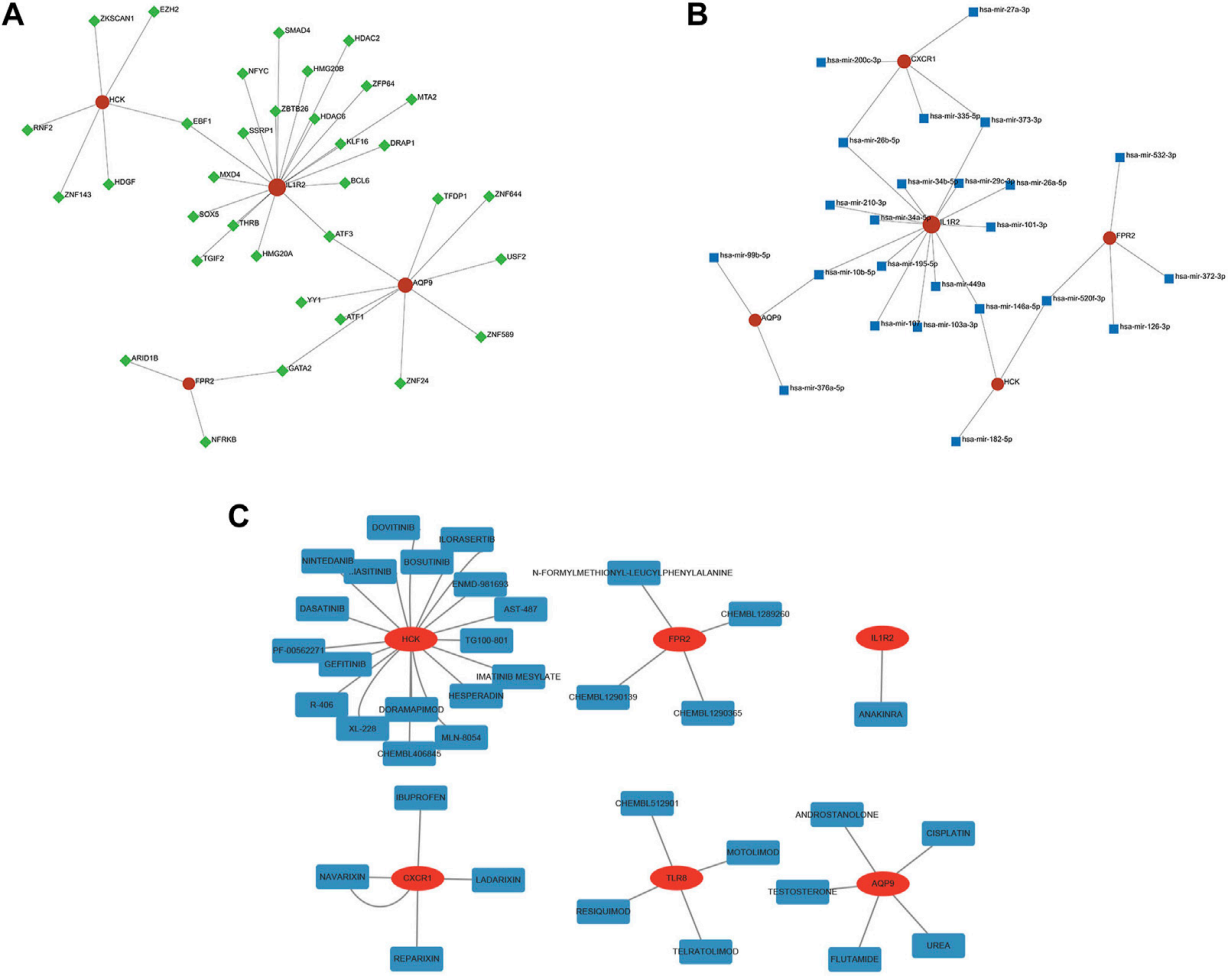
TF-, miRNA-, and drug-gene network of the diagnostic genes. **(A)** The TF network of HCK, IL1R2, AQP9, and FPR2. **(B)** The miRNA network of CXCR1, HCK, IL1R2, AQP9, and FPR2. **(C)** The drug-gene network of six diagnostic genes.

We used the NetworkAnalyst database to predict the TFs that may regulate diagnostic genes and generated a TF-diagnostic gene network (Figure 10A). The network consisted of 4 diagnostic genes and 34 TFs. Then the NetworkAnalyst database was used to predict related miRNAs and formed a regulatory network diagram (Figure 10B). This regulatory network diagram contained 5 diagnostic genes and 24 miRNAs, the most abundant of which were regulating IL1R2.

Finally, the DGIdb database was utilized and made it possible to search for potential drugs that might treat IVIG resistance. We found 36 potential therapeutic drugs and 11 of them were approved (Figure 10C; Supplementary Table S4). These results helped to deepen our understanding of the regulatory and intervention mechanisms of these diagnostic genes and provided clues for further research.

## 4 Discussion

Kawasaki disease is an acute self-limiting febrile illness of unknown etiology that primarily affects children under 5 years of age (McCrindle et al., 2017). Prompt treatment of KD patients with IVIG has been shown to reduce the incidence of coronary artery aneurysms from 25% to less than 5% (Altammar and Lang, 2018). However, approximately 10%–20% of KD patients experience persistent or recurrent fever after standard treatment with IVIG and aspirin (Tremoulet et al., 2008; Tremoulet et al., 2014). Patients with IVIG resistance are at higher risk of developing coronary artery aneurysms (Hamada et al., 2019). Risk factors associated with IVIG resistance include male sex, young age, high C-reactive protein levels, high neutrophil count, and early presentation, among others (Lo and Newburger, 2018). Although there are currently some risk scoring systems that can predict the responsiveness of IVIG, they are not so accurate enough that no scoring systems are universally accepted (Wang et al., 2019). Recent studies have shown that immune cell infiltration is one of the important factors in the development of IVIG resistance in KD, but only a few studies have linked IVIG responsiveness to biomarkers related to the level of immune cell infiltration (Wu et al., 2020). Therefore, we aimed to explore the immunogenetic mechanisms of IVIG resistance in KD to screen for potential diagnostic indicators and signaling pathways in those patients. This study will help to better understand the pathogenesis and treatment of IVIG resistance and ultimately provide patients with more optimal treatment options.

This study explored the differences in immune cell infiltration between IVIG-resistant and IVIG-responsive patients by the CIBERSORT algorithm and found that neutrophil infiltration had significant differences in the degree of infiltration. Similar results were reported in previous studies, indicating the important role of neutrophils in the pathogenesis of IVIG resistance. Studies showed that compared to IVIG-responsive patients, IVIG-resistant patients had a higher proportion of neutrophils, a higher neutrophil-to-lymphocyte ratio (NLR), and NLR was also considered to be a reliable predictor for IVIG resistance (Ha et al., 2020; Y. Chen et al., 2019; Liu and Wu, 2022). It could be seen that infiltrating immune cells were closely related to IVIG resistance, and the role of neutrophils was indispensable in the mechanism.

Next, the gene module with the highest score in the module-trait relationship diagram was screened through WGCNA analysis, which was the most correlated with neutrophil expression. By intersecting the module genes with the DEIGs, 34 DENGs were obtained. KEGG enrichment analysis showed that they were mainly concentrated in pathways such as cytokine-cytokine receptor interaction, neutrophil extracellular trap formation, and coronavirus disease - COVID-19, indicating their important biological functions in immune response and regulation. It is noteworthy that neutrophils contribute to pathogen clearance by forming neutrophil extracellular traps (NETs) in a process known as NETosis, but the excessive release of NETs has been reported to be involved in the pathogenesis of various diseases, including vasculitis, by inducing tissue injury (Grayson and Kaplan, 2016). In KD patients, neutrophils increase from the acute phase and decrease in the recovery phase; studies have shown that NET formation is enhanced in acute KD patients compared to recovery-phase KD patients and healthy controls (Yoshida et al., 2020). In addition, it has been found that neutrophils are significantly increased in the acute phase of KD patients, and are prone to forming NETs, which can significantly promote the activation of PI3K/Akt and NF-κB signaling pathways in peripheral blood mononuclear cells (PBMCs) of KD patients, leading to upregulation of HIF-1α and VEGF expression and triggering more severe inflammatory responses (Jing et al., 2020). This indicates that NETs are one of the mechanisms of Kawasaki disease, however, whether NETs can distinguish between IVIG-resistant and IVIG-responsive patients has not been proven by previous research. Our data analysis results showed that the expression of neutrophils in the two groups of patients is significantly different, and the enriched pathway of differentially expressed genes contained NETs. Therefore, we believed that NET formation was expected to be an important mechanism of IVIG resistance. In addition, the pathway of coronavirus disease - COVID-19 is also closely related to neutrophils. It is reported that the number of neutrophils is considered as a clinical marker related to acute respiratory distress syndrome in COVID-19 patients (Oishi et al., 2022). Moreover, one study has also demonstrated that similar neutrophilic dysregulation occurs in severe COVID-19 and KD two pronounced hyperinflammatory states, which plays a crucial role in the overactivation and defective aging program of granulocytes (Chen K D et al., 2022). In short, based on the results of the KEGG enrichment analysis, we could conclude that these genes were more likely to be involved in neutrophil-related biological activities.

Finally, six hub genes, including TLR8, AQP9, CXCR1, FPR2, HCK, and IL1R2, were identified from the 34 DENGs by the MCODE algorithm. These hub genes may serve as diagnostic biomarkers for distinguishing IVIG-resistant patients from IVIG-responsive patients based on ROC curve analysis. Further investigation was conducted to explore the functions of each hub gene. TLR8, a member of the Toll-like receptor (TLR) family, is an intracellular type I transmembrane protein primarily expressed in human monocytes, macrophages, and neutrophils (Cai and Hu, 2022). Studies have indicated that TLR8 expression increases as a marker of M2 macrophages during the acute phase of KD (Guo et al., 2020). Aquaporins (AQPs) are transmembrane channels essential for water, energy, and redox homeostasis, with proven involvement in various pathophysiological conditions. AQP9 has been reported to be upregulated in patients with systemic inflammatory response syndrome compared to healthy individuals, and this is attributed to the functional impact of AQP9 on F-actin polymerization, leading to changes in the morphology and function of neutrophils (da Silva et al., 2022). CXC chemokine receptor-1 (CXCR1) is a representative chemokine receptor that initiates the neutrophil-mediated immune response pathway through its association with its homologous chemokine interleukin-8 (IL-8 or CXCL8) and can control the migration of neutrophils to infected tissues (Kharche et al., 2021). The formyl peptide receptors (FPRs) are G protein-coupled receptors, and formyl peptides act on FPR1 and FPR2, transducing chemotactic signals in phagocytes, and mediating host-defense as well as inflammatory responses including cell adhesion, directed migration, granule release, and superoxide production (He and Ye, 2017). It has been shown that the expression of FPR2 in the KD group is significantly higher than that in the healthy control group, and the increase in FPR2 levels may be involved in the regulation of the *in vivo* balance of the immune system (Wang Y et al., 2022). Hematopoietic cell kinase (HCK) is a member of the Src family of non-receptor tyrosine kinases, which plays a critical role in neutrophil phagocytosis (Cheng et al., 2022). Studies have shown that HCK is a significantly upregulated hub gene in KD, but its specific mechanism of action is not yet clear (Cai and Hu, 2022). Interleukin-1 receptor type II (IL1R2) is a member of the interleukin-1 (IL-1) receptor family and is abnormally expressed in many inflammatory diseases (Chen Q et al., 2022). Studies have shown that IL1R2 is upregulated in IVIG-resistant KD, which is consistent with our research conclusion (Geng et al., 2020). Seen from the above gene functions, they are all related to the body’s immune response and are highly correlated with neutrophils. This was also confirmed by Spearman’s correlation analysis in our study.

What’s more, we predicted the TFs and miRNAs that regulate the hub genes through the NetworkAnalyst database, then searched for potential therapeutic drugs by the DGIdb database, to further explore the regulatory mechanisms and intervention methods of these hub genes. Because these results were derived from continuously updated resources from papers, databases, and web resources that can provide directions for further research, the reliability of some of these predicted molecules will require experimental proof.

Despite yielding interesting results, there are some limitations in our study. Firstly, this study covered a relatively small sample size, and a larger sample cohort is needed to confirm the research results. Secondly, the lack of experimental validation may affect the accuracy of the study, so further experiments are needed to verify the biological mechanisms and treatment response. In addition, since our study only analyzed samples of KD patients who developed resistance or response to IVIG after use, it cannot be inferred that these genes can be used as diagnostic genes for IVIG resistance before treatment. Therefore, adopting KD patient samples that have already been diagnosed with IVIG resistance or response and analyzing their genes before IVIG use may help to identify IVIG resistance earlier and improve treatment plans as soon as possible.

All in all, the results of this study suggest new clues for the potential pathogenesis of IVIG resistance and provide valuable insights into the diagnosis and treatment of IVIG resistance. Previous studies have shown that the percentage of neutrophils in leukocytes, percentage of polymorphonuclear neutrophils, peripheral blood neutrophil-to-lymphocyte ratio, and neutrophil activation rate are higher in the IVIG-resistant group relative to the IVIG-responsive group (Sato et al., 2013; Kawamura et al., 2016; Kim, Song, and Kim, 2018; Ko et al., 2019). Our study found that immune pathways involved in hub genes, such as the formation of neutrophil extracellular traps, were more likely to result in IVIG resistance. And these hub genes, TLR8, AQP9, CXCR1, FPR2, HCK, and IL1R2, verified to be more or less associated with neutrophils, were possible to lead to IVIG resistance in KD. Therefore, our results corroborated previous literature and no longer stopped at clinical indicators, but explored more deeply at the genetic level. We believed that they may serve in the future as a more in-depth complement to the diagnostic model of IVIG resistance clinical indicators and important therapeutic targets that may help to reduce the incidence of coronary artery abnormalities and to improve the quality of life of KD patients.

## 5 Conclusion

In this study, we investigated potential signaling pathways and hub genes associated with immune infiltration involved in IVIG resistance, and the final identified hub genes included TLR8, AQP9, CXCR1, FPR2, HCK, and IL1R2. Additionally, we found that these signaling pathways and hub genes were closely related to neutrophils, which was confirmed by previous studies and Spearman’s correlation analysis in our study. And six hub genes were validated that they could serve as reliable diagnostic biomarkers for IVIG resistance. Finally, their related TFs, miRNAs, and targeted drugs were predicted, which would provide new ideas and methods for further understanding the mechanism of IVIG resistance and help develop treatment strategies.

## Data Availability

The original contributions presented in the study are included in the article/Supplementary Material, further inquiries can be directed to the corresponding authors.
