# Supplementary material for: Identification of key signaling pathways and hub genes related to immune infiltration in Kawasaki disease with resistance to intravenous immunoglobulin based on weighted gene co-expression network analysis
Source: Front Mol Biosci. 2023 May 30;10:1182512. doi: 10.3389/fmolb.2023.1182512 (PMC10267737; doi:10.3389/fmolb.2023.1182512)
Supplement: Supplementary file 1 [file DataSheet2.ZIP › Supplementary Materials/Supplementary Table Legends.pdf]

Supplementary Material

Supplementary Table Legends

Supplement Table 1. All DEGs, upregulated DEGs, and downregulated DEGs in two groups.

Supplement Table 2. The intersection of DEGs and IGs.

Supplement Table 3. The intersection of genes in key module (blue module) and DEIGs.

Supplement Table 4. The potential drugs targeting diagnostic genes.
